# Fast and Robust Infrared Small Target Detection Using Weighted Local Difference Variance Measure

**DOI:** 10.3390/s23052630

**Published:** 2023-02-27

**Authors:** Ying Zheng, Yuye Zhang, Ruichen Ding, Chunming Ma, Xiuhong Li

**Affiliations:** Key Laboratory of Signal Detection and Processing, Department of Information Science and Engineering, Xinjiang University, Urumqi 830017, China

**Keywords:** infrared (IR) small target, new tri-layer filtering window, local difference variance measure (LDVM), weighting function, window intensity level (WIL)

## Abstract

Infrared (IR) small-target-detection performance restricts the development of infrared search and track (IRST) systems. Existing detection methods easily lead to missed detection and false alarms under complex backgrounds and interference, and only focus on the target position while ignoring the target shape features, which cannot further identify the category of IR targets. To address these issues and guarantee a certain runtime, a weighted local difference variance measure (WLDVM) algorithm is proposed. First, Gaussian filtering is used to preprocess the image by using the idea of a matched filter to purposefully enhance the target and suppress noise. Then, the target area is divided into a new tri-layer filtering window according to the distribution characteristics of the target area, and a window intensity level (WIL) is proposed to represent the complexity level of each layer of windows. Secondly, a local difference variance measure (LDVM) is proposed, which can eliminate the high-brightness background through the difference-form, and further use the local variance to make the target area appear brighter. The background estimation is then adopted to calculate the weighting function to determine the shape of the real small target. Finally, a simple adaptive threshold is used after obtaining the WLDVM saliency map (SM) to capture the true target. Experiments on nine groups of IR small-target datasets with complex backgrounds illustrate that the proposed method can effectively solve the above problems, and its detection performance is better than seven classic and widely used methods.

## 1. Introduction

Infrared (IR) imaging technology has been widely used in civilian fields such as car navigation, diseased-cell diagnosis, industrial-flaw detection, physiological performance of animal life processes, and plant monitoring [1]. It is worth noting that the infrared search and track (IRST) system based on IR imaging technology has the advantages of passive surveillance, all-weather use, and high spatial resolution, and uses the difference in thermal radiation between the target and the background to achieve long-distance target detection [2,3]. It has very important application value in military fields such as precision guidance, early-warning systems, space-based surveillance, and geological analysis [4,5]. IR small-target detection plays a vital role in these applications. To find the target as early as possible, long-distance detection and tracking are required, so the target has few pixel and texture features and lacks shape and structure information [6]. Furthermore, targets are usually immersed in complex backgrounds, and targets can be affected by a low signal-to-clutter ratio (SCR) [7]. Therefore, IR small-target detection is still a difficult and challenging task.

IR small-target detection methods in complex scenes can be divided into sequence detection methods and single frame detection methods [8,9]. Compared with the sequence detection method, the single frame detection method has a small amount of calculation and strong scene adaptability. Since real-time target detection becomes urgent in the military application of an IRST system, research based on single frame detection method is very necessary [10,11].

Existing single-frame detection methods can be divided into four categories. The first category is based on filtering methods, which are divided into algorithms based on spatial filtering and algorithms based on frequency-domain filtering. The algorithm based on spatial filtering is simple in design, fast in calculation speed, and has better performance in a uniform background, but it is easy to cause false detection in a complex background and has poor robustness [12,13]. Although algorithms based on frequency-domain filtering can suppress complex backgrounds, they have high computational complexity [14,15]. The second category is based on low-rank sparse restoration methods, which have high detection performance under strong noise background conditions, but have high computational complexity when dealing with large-scale images [16,17,18,19]. The third category is methods based on deep learning, which can improve the detection accuracy of small targets to a certain extent, but lack many datasets in various forms, which is challenging [20,21,22,23,24]. The fourth category is methods based on the human visual system. This system is relatively real-time and it is not easy to lose target features during the detection process and, but it is easy to cause false positives in complex scenes [25,26,27,28,29,30,31,32,33,34,35]. Given the importance of real-time detection and detection rate, this paper was inspired by the human visual system, a brief overview of detection methods based on the human visual system follows.

IR small-target detection algorithms based on the local-contrast method of the human visual system have attracted much attention. These algorithms focus on the differences between the target and the background surrounding it. For instance, Chen et al. [5] proposed a local contrast measure (LCM) that uses nested windows with eight orientations to suppress background edges; Han et al. [25] proposed an improved LCM (ILCM) that uses the target area average to suppress pixel-sized noise with high brightness (PNHB); Han et al. [26] proposed the relative LCM (RLCM) computed by combining ratio differences, and then generalizing it to the sub-block level [27]; Wei et al. [28] used the multi-scale patch-based contrast measure (MPCM) algorithm to fuse the corresponding two directions into a whole to capture the target; Han et al. [29] adopted a multi-scale three-layer local contrast measure (TLLCM), used Gaussian filtering to enhance the target area, and took the average value of several largest pixels in the surrounding area; Moradi et al. [30] proposed absolute directional mean difference (ADMD), which uses an orientation method to suppress the structural background; and Zhang et al. [20] proposed a multi-scale strengthened directional difference (MSDD) algorithm, which combines the local directional-intensity measure and the local directional-fluctuation measure to effectively suppress the angular clutter. Furthermore, in existing studies, many researchers are keen to employ weighting functions on top of basic local-contrast algorithms to improve detection performance. For example, Qin et al. [10] used the variance of the central unit as the weight function; Deng et al. [31] improved the local entropy as the weight function; Nasiri et al. [32] used the center and surrounding variance difference (VAR_DIFF) as the weighting function; Liu et al. [33] proposed a weighted LCM, which defines a weighting function based on the strong clutter edge features; Lv et al. [34] proposed the regional intensity level (RIL) algorithm to assess the complexity level of each unit, taking the RIL difference between the central unit and its surrounding background as a weighting function; and Han et al. [35] proposed weighted strengthened LCM (WSLCM) and proposed an improved RIL (IRIL) that replaces the maximum with the average of several maximum grayscale calculations. 

The weighted LCM using more local information can reduce the false-alarm rate to a certain extent. However, there are still some problems. First, current algorithms usually directly compute the contrastive information between the target area and surrounding areas, but when the target scale is small, the edge information cannot be captured for effective enhancement. Second, some weighting algorithms increase the time of image processing during detection. Third, the existing methods do not sufficiently consider the shape of the true target, and the detection process is easily disturbed by noise.

To better enhance targets of different scales in different complex scenes, ensure a certain detection time, better preserve target shape characteristics, and reduce false-alarm rates, a detection framework based on weighted local difference variance measure (WLDVM) is proposed. First, the image is preprocessed by Gaussian filtering, and then according to the distribution characteristics in the target area, the target area is divided into a new tri-layer filtering window and the window intensity level (WIL) value of each layer of windows is calculated. Second, the local difference variance measure (LDVM) and weighting function are calculated by ratio and difference operations using the obtained position and WIL value of each layer window. Finally, a simple threshold is used to segment the fused result WLDVM to capture the true target. The contributions of this paper are as follows:

1. The new tri-layer filtering window is proposed. The target area is divided according to its distribution characteristics and size, which can adapt to the detection of targets of different scales and save detection time.
2. WIL is proposed. Each layer of window uses the mean of the two largest subblock averages instead of the single largest subblock average to better capture the target and suppress edge noise.
3. LDVM is proposed. Through the idea of local fluctuation, the target area is further enhanced, and the high-brightness background is eliminated.
4. A detection framework based on WLDVM is proposed. The experimental results using multiple sets of IR datasets show that the proposed algorithm has the best detection performance and consumes less time.

## 2. Proposed Algorithm

**Figure 1:**
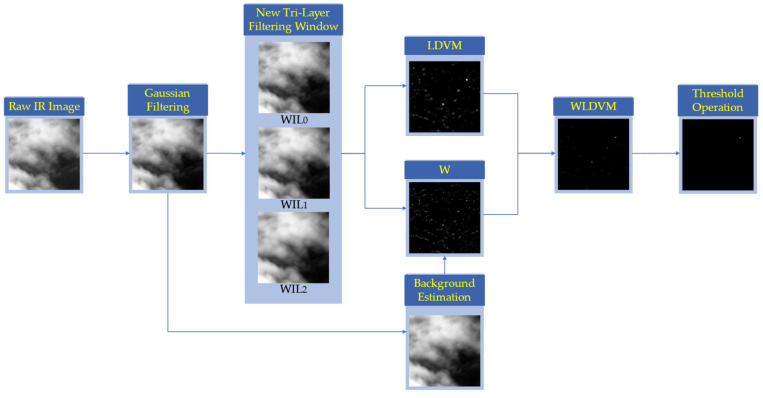
The proposed algorithm framework.

Figure 1 shows the proposed WLDVM algorithm framework. First, the image is preprocessed by Gaussian filtering, and the WIL values of each layer are calculated through the new tri-layer filtering window. Then, according to the WIL value and location of each layer, the idea of local fluctuation and background estimation is introduced to calculate LDVM and weighting function. The true small target is the most prominent in the final weighted result, which can be easily captured with a simple threshold segmentation.

### 2.1. Gaussian Filtering Pre-Processing

Small targets in IR image usually have a low SCR and are susceptible to noise interference because of the effects of long-distance and atmospheric transmission. These factors make detection more difficult, requiring noise suppression and target enhancement. The best filter for improving the target should have the same distribution as the target, according to the matched filter theory [36] and given that small IR targets have Gaussian-like properties and that Gaussian filters are excellent at suppressing high-frequency IR image components including scattered noises, Gaussian noises, and PNHB [29,37]. In this study, noise is reduced and small targets are enhanced using Gaussian filtering. The result of the Gaussian filtering operation is expressed as

(1) $$GI\left(x,y\right)=\sum_{l=-1}^{1}\sum_{k=-1}^{1}G\left(l,k\right)I\left(x+l,y+k\right),\;G=\frac{1}{16}\left[\begin{array}{c} 1\;\;2\;\;1\\ 2\;\;4\;\;2\\ 1\;\;2\;\;1 \end{array}\right].$$

where G is the Gaussian template and I is the original IR image.

### 2.2. Construction of the New Tri-Layer Filtering Window

**Figure 2:**
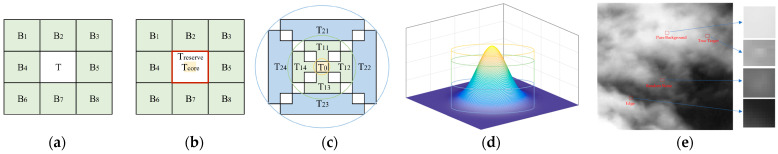
(**a**) The double-layer window. (**b**) The tri-layer window. (**c**) The new tri-layer filtering window. (**d**) Gaussian distribution characteristics of IR small target. (**e**) Situations that the algorithm needs to handle.

Traditional LCM and its improved algorithms adopt a double-layer filtering window, the central unit captures the target area, and the surrounding units capture the background area around the target, see Figure 2a. But when the scale of the true target area is smaller than the central unit scale, the detected target will be enlarged. Therefore, Nasiri et al. [32] made an improvement and proposed a three-layer nested window to divide the central unit into two parts, namely the core layer and the reserve layer. The core layer captures the main energy of the target area, and the reserve layer separates the target from its surrounding units, see Figure 2b. Usually, PNHB in complex backgrounds is difficult to suppress because its core layer differs significantly from surrounding layers.

It is well known that the real target area has a compact two-dimensional Gaussian shape distribution whose intensity weakens towards the surroundings, as shown in Figure 2d, while PNHB does not possess such a distribution. In this paper, according to the target area distribution characteristics in Figure 2d, a new tri-layer filtering window is proposed to capture the target area, namely inner layer ($T_{0}$ yellow area), middle layer ($T_{1}$ green area), and outer layer ($T_{2}$ blue area), see Figure 2c. According to SPIE, the total spatial extent of the small target is usually less than 80 pixels [5]. Therefore, the inner layer is set to 1 × 1; through four directions and the middle layer and the outer layer are each divided into four subblocks. The subblock of the middle layer is a symmetrical trapezoid with a height of 2, an upper base of 1, and a lower base of 3. The subblock of the outer layer is a symmetrical trapezoid with a height of 2, an upper base of 5, and a lower base of 7. The proposed new tri-layer filtering window can adapt to the detection of targets of different scales, and its total space is small, which will make the algorithm run faster.

### 2.3. Calculation of the Window Intensity Level (WIL)

Apply the new tri-layer filtering window from top to bottom and left to right on the Gaussian filtered image and follow the steps below to calculate the WIL value for each layer of each pixel.

1. For the inner layer:

(2) $${\mathrm{WIL}}_{T_{0}}=GI_{T_{0}}$$

where $GI_{T_{0}}$ is the pixel in cell $T_{0}$ of the Gaussian filtered image GI.

2. For the middle and outer layers:

First, the average value of each subblock in the layer is calculated as the key parameter for the next calculation:

(3) $$M_{T_{ij}}=\frac{1}{N_{T_{ij}}}\;\sum_{k=1}^{N_{T_{ij}}}GI_{T_{ij}}^{k}i=1,2;j=1,2,3,4$$

where $N_{T_{ij}}$ is the total number of pixels in cell $T_{ij}$, and $GI_{T_{ij}}^{k}$ is the gray value of the kth pixel in cell $T_{ij}$.

${\mathrm{WIL}}_{T_{i}}$ is the mean of the m largest $M_{T_{ij}}$ values in $T_{i}$ area, that is,

(4) $${\mathrm{WIL}}_{T_{i}}=\frac{1}{m}\sum_{l=1}^{m}M_{T_{i}}^{l}i=1,2;m=2$$

where $M_{T_{i}}^{l}$ is the lth largest $M_{T_{ij}}$ value in the $T_{i}$ area. The distribution trend of the cloud layer is a gradual process; the interior of the cloud layer changes slowly, and the gray value of the edge fluctuates greatly, as shown in Figure 2e. With this type of edge it is easy to cause the occlusion of the weak target, and the gray value of the inner layer of the small target at the cloud edge is at least not much different from the average gray value of a sub-block of other layers. To effectively enhance this type of small target to avoid missed detection, m in Equation (4) needs to be greater than 1. When m is greater than 2, edge clutter will be enhanced to cause false alarms, so 2 is the most suitable value for m.

### 2.4. Local Difference Variance Measure (LDVM)

The local contrast in the form of differences can eliminate the high-brightness background. The difference of WIL is defined by the difference between layers as

(5) $$DoWIL=\{\begin{array}{cc} {\mathrm{WIL}}^{T_{q}}-{\mathrm{WIL}}^{T_{p}} & ,\;p>q\\ 0 & ,\mathrm{others} \end{array}$$

where ${\mathrm{WIL}}^{T_{q}}$ indicates that the maximum value in ${\mathrm{WIL}}_{T_{i}}$ is in the $T_{q}$ layer and ${\mathrm{WIL}}^{T_{p}}$ indicates that the minimum value in ${\mathrm{WIL}}_{T_{i}}$ is in the $T_{p}$ layer. Clutter can be further suppressed by non-negative constraints.

Through the above calculation, there are cases where pixels are suppressed at the edges inside the target area. To prevent these pixels from being suppressed by further calculations, this paper enhances areas with large local fluctuations by computing the mean filtering of the square of the image minus the square of its mean filtering. The LDVM of each pixel is defined as

(6) $$\mathrm{LDVM}\left(x,y\right)=M_{2L}\left(x,y\right)-{\left(M_{L}\left(x,y\right)\right)}^{2}$$

where $M_{2L}$ and $M_{L}$ are defined as

(7) $$M_{2L}\left(x,y\right)=\sum_{l=-2}^{2}\sum_{k=-2}^{2}MF\left(l,k\right){\left(DoWIL\left(x+l,y+k\right)\right)}^{2}$$

(8) $$M_{L}\left(x,y\right)=\sum_{l=-2}^{2}\sum_{k=-2}^{2}MF\left(l,k\right)DoWIL\left(x+l,y+k\right)$$

where MF is a 5 × 5 normalized mean filtering template. Obviously, the gray value of the local area of the pixels at the edges inside the target area fluctuates greatly, so these pixels are effectively enhanced.

### 2.5. Weighting Function

The local contrast in the form of ratio can enhance the true target. The ratio of WIL is defined by the difference between layers as

(9) $$RoWIL\left(x,y\right)=\{\begin{array}{cc} {\mathrm{WIL}}^{T_{q}}/{\mathrm{WIL}}^{T_{p}} & ,\;p>q\\ 0 & ,\mathrm{others} \end{array}.$$

Mean filtering can reduce the sharp change of image gray value to achieve the purpose of smoothing the image. In this paper, the background estimation is performed by mean filtering as

(10) $$BE\left(x,y\right)=\sum_{l=-2}^{2}\sum_{k=-2}^{2}MF\left(l,k\right)GI\left(x+l,y+k\right)$$

Although RoWIL as an enhancement factor can effectively enhance the target area, there is still a lot of background clutter. In this paper, background estimation is used to calculate the weight function of each pixel in the form of ratio difference combination to suppress part of the background clutter, which is defined as

(11) W(x,y)=max{0,RoWIL(x,y)GI(x,y)−BE(x,y)}

In general, the weight of the true target is very large, and its surrounding local background is completely suppressed, so the weighting function fully considers the shape of the target.

### 2.6. Weighted Local Difference Variance Measure (WLDVM)

The LDVM and weighting function are fused to obtain the WLDVM of the current pixel, that is

(12) WLDVM(x,y)=W(x,y)LDVM(x,y).

The calculation of LDVM can better eliminate the high-brightness background and make the whole target appear high-brightness. The operation result of the weighting function can fully consider the shape of the target. The WLDVM algorithm preserves the shape of the original target, the target is effectively enhanced, and the background is effectively suppressed. In most cases the target size is unknown and multi-scale detection is required. In this paper, multi-scale detection is not required, and efficient detection can be performed, which greatly saves detection time.

### 2.7. Threshold Operation

The saliency map (SM) of each IR image can be obtained by computing WLDVM and the different results produced by the pixels of different situations are analyzed.

1. For a pixel in the real target area, since the target area often presents a compact two-dimensional Gaussian shape, its DoWIL will be large and RoWIL>1, and its LDVM and weight will be very large. Hence, the resulting value of WLDVM will be large.
2. For a pixel in the pure background area, since the pure background area is often continuous and evenly distributed, its DoWIL≈0 and RoWIL≈1, then its LDVM≈0 and W≈0. Therefore, WLDVM≈0.
3. For a pixel at the edge of the background, its DoWIL may be greater than 0 but less than that of the true target, so its LDVM is much less than that of the true target; in addition, RoWIL may be greater than 1, but its enhancement effect is not much different from the local background estimation, so the corresponding W will be less than the true target’s W. Hence, its WLDVM is much less than that of the true target.
4. For a pixel in the PNHB area, its DoWIL will be less than that of the true target, and thus its LDVM will be less than the true target’s LDVM; in addition, its W will be less than the true target’s W. Hence, its WLDVM is much less than that of the true target.

As can be seen from this discussion, the true target area will be the most salient in SM, so a simple threshold operation is used to extract it, the threshold is defined as

(13) $$Th=\lambda max_{SM}+\left(1-\lambda \right)mean_{SM}$$

where $max_{SM}$ is the maximum gray value of SM, and $mean_{SM}$ is the average gray value of SM. λ is an experimental constant between 0 and 1. In the experimental part, the value of λ is analyzed in detail, and the experiment shows that λ can take any value between 0.5 and 0.6.

## 3. Experimental Results

To demonstrate the detection performance of the proposed algorithm, nine groups of IR datasets were used, including three sets of real IR sequences (datasets 1, 3, and 4), five sets of simulated IR sequences (datasets 2, 5, 6, 7, and 8), and one non-sequential dataset (dataset 9). Datasets are shown in [5,38,39,40,41,42,43]. The targets in dataset 1 are all immersed in very complex dense cloud cover and most of the targets have very low contrast. The targets in dataset 2 are all immersed in a dimly lit background. The target in dataset 3 moves from the cloud layer to the cloudless area, some targets have low contrast, and the background contains a lot of noise. The aircraft target in dataset 4 is large in scale and immersed in a cloudless area with a few thin clouds in the background. The target in dataset 5 moves from a cloudless dark background area into thin cloud cover. The target in dataset 6 is immersed in a complex air and sea background, which contains many PNHBs. Targets in dataset 7 move from a background containing buildings. The targets in dataset 8 are immersed in complex and changing land backgrounds. Dataset 9 consists of representative images of different sequences, with both targets and backgrounds differing between images. Additional details are shown in Table 1.

First, we analyzed the effect of λ value on detection performance. Table 2 shows the number of false-alarm images $N_{FA}$ and the number of missed images $N_{MD}$ corresponding to different datasets under different values of λ, where λ increases from 0 to 1 with a step size of 0.1. The experiments showed that when the value of λ was between 0.5 and 0.6, small targets in different complex scenes could be effectively captured, there were no missed detections and false alarms in any dataset, and high classification accuracy could be obtained.

Then, seven LCM-based algorithms were selected from multiple perspectives for comparison with the proposed algorithm, including LCM [5], MPCM [28], RLCM [26], TLLCM [29], VAR_DIFF [32], ADMD [30], and WSLCM [35]. Among them, VAR_DIFF and TLLCM are local-contrast algorithms based on tri-layer windows, and the rest are local-contrast algorithms based on double-layer windows; RLCM, TLLCM, and WSLCM are local-contrast methods using ratio difference joint operations; and VA_DIFF and WSLCM are local-contrast algorithms that use the weighting function.

**Figure 3:**
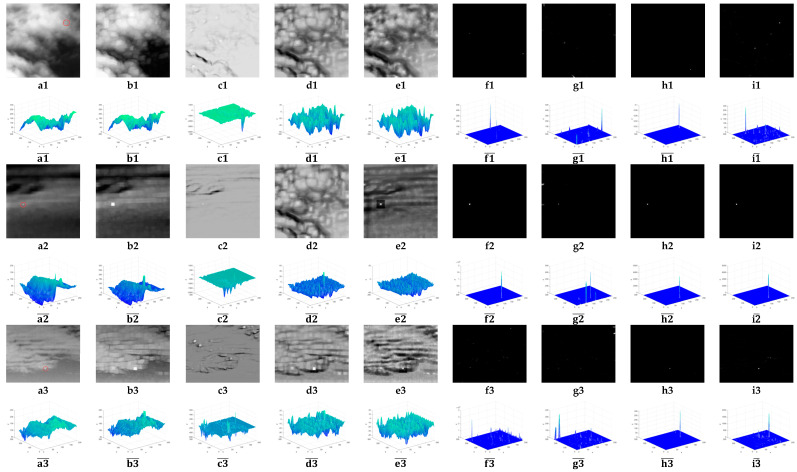

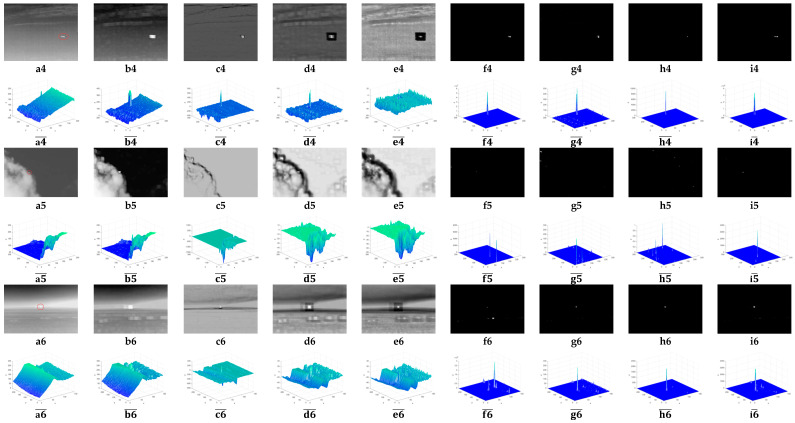

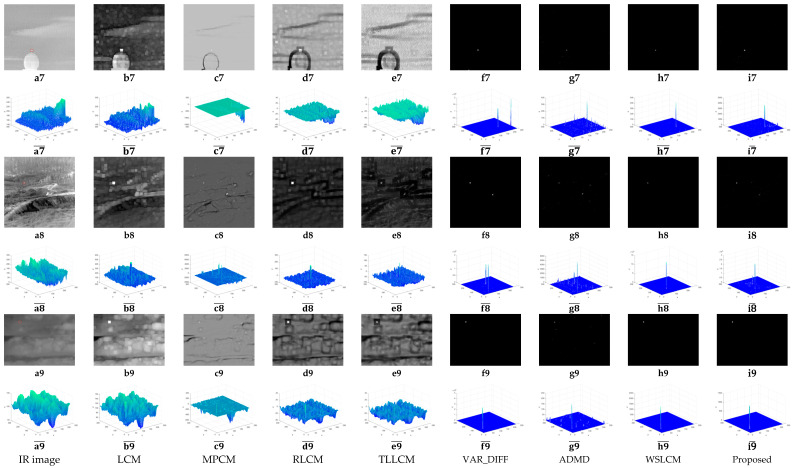
SMs of different algorithms. (**a1**–**a9**) Nine representative IR images. (**b1**–**b9**) LCM. (**c1**–**c9**) MPCM. (**d1**–**d9**) RLCM. (**e1**–**e9**) TLLCM. (**f1**–**f9**) VAR_DIFF. (**g1**–**g9**) ADMD. (**h1**–**h9**) WSLCM. (**i1**–**i9**) Proposed methods. ($\bar{a1}–\bar{a9}$), ($\bar{b1}–\bar{b9}$ ), ($\bar{c1}–\bar{c9}$ ), ($\bar{d1}–\bar{d9}$ ), ($\bar{e1}–\bar{e9}$ ), ($\bar{f1}–\bar{f9}$ ), ($\bar{g1}–\bar{g9}$ ), ($\bar{h1}–\bar{h9}$ ), and ($\bar{i1}\text{–}\bar{i9}$ ) are the 3D gray distribution maps of (**a1**–**a9**), (**b1**–**b9**), (**c1**–**c9**), (**d1**–**d9**), (**e1**–**e9**), (**f1**–**f9**), (**g1**–**g9**), (**h1**–**h9**), and (**i1**–**i9**), respectively.

To analyze different methods intuitively, Figure 3 shows the SMs of different algorithms. Each dataset’s original image sample may be found in the first column. The target size was variable, the backdrop was intricate, and there were various levels of noise present. As shown in the second column of the figure, LCM enhanced the target and made the target area larger, while enhancing the noise, and the background suppression effect was not good. As shown in the third column of the figure, MPCM enhanced the target but did not preserve the target shape very well and had a certain suppression effect on the background and noise, but when the background was more complex, the detection effect was not good. As shown in the fourth column of the figure, RLCM enhanced the target and made the target area larger and had a general effect on background and noise suppression. As shown in the fifth column, TLLCM had a mediocre level of noise and background suppression efficiency, but the detection effect was poor when the background was complicated. As shown in the sixth, seventh, and eighth columns of the figure, VAR_DIFF, ADMD, and WSLCM had better background suppression effects, but when the background was complex, the noise suppression effect was average, and the detection performance was unstable. As shown in the ninth column of the figure, the proposed method effectively improved the target SCR and better preserved the target outline, could better suppress the background and noise, and the detection performance was the best.

To illustrate the detection performance of these algorithms, the indicator’s signal-to-clutter ratio gain (SCRG) and background suppression factor (BSF) before thresholding are used simultaneously, and defined as

(14) $$SCRG=\frac{SCR_{out}}{SCR_{in}},\;BSF=\frac{\sigma_{in}}{\sigma_{out}}\;,\;SCR=\frac{\left|m_{t}-m_{b}\right|}{\sigma_{b}}.$$

where $SCR_{in}$ is the SCR value of the original image, $SCR_{out}$ is the SCR value of the SM, $\delta_{in}$ is the standard deviation of the non-target area in the original image and $\delta_{out}$ is the standard deviation of the non-target area in the SM, and $m_{t}$ is the mean of the target area, $m_{b}$ and $\sigma_{b}$ are the mean and standard deviation of the local background area around the target, respectively. It can be seen in Table 3 that VAR_DIFF had one set with the highest SCRG value, WSLCM had two sets with the highest SCRG value, and the proposed algorithm had six sets with the highest SCRG value and the highest average SCRG value. The results show that the proposed method achieved more significant target enhancement before thresholding than other methods. VAR_DIFF had one set with the highest BSF value, WSLCM had five sets with the highest BSF value, and the proposed algorithm had three sets with the highest BSF value and the highest mean. It shows that the background-suppression ability of the proposed algorithm is equivalent to that of the WSLCM algorithm, and better than that of other algorithms.

**Figure 4:**
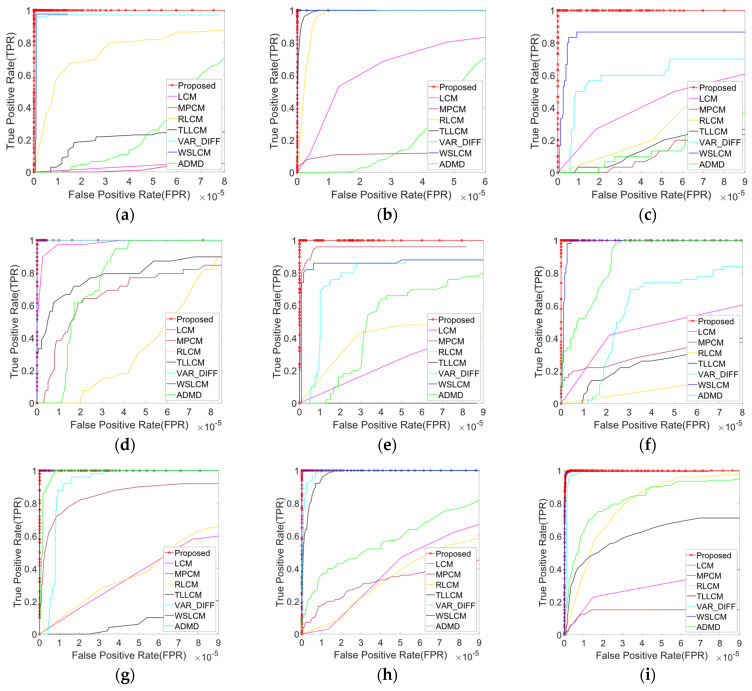
The ROC curves of different detection methods. (**a**−**i**) The experimental results of datasets 1−9.

Figure 4 depicts the receiver operating characteristic (ROC) curves for different algorithms to evaluate the target-enhancement ability and background-suppression ability after thresholding, where the false-positive rate (FPR) and the true-positive rate (TPR) are the horizontal and vertical coordinates of the ROC curve [44], respectively, and are defined as

(15) $$\mathrm{FPR}=\frac{N_{false}}{N_{pixel}}\;,\;\mathrm{TPR}=\frac{N_{detected}}{N_{ture}}\;.$$

where $N_{false}$ is the number of detected false targets, $N_{pixel}$ is the total number of pixels in the whole image, $N_{detected}$ is the number of detected true targets, and $N_{ture}$ is the total number of true targets.

In the ROC curve, the more the curve shifts to the upper left corner, the better the detection performance will be. Under the same FPR, the larger the TPR, the better the performance of the algorithm. As can be seen from Figure 4, when $\mathrm{FPR}=10^{-5}$:

- LCM had a TPR greater than 0.9 and less than 1 in dataset 4, and performed poorly in other datasets;
- MPCM had a TPR greater than 0.9 and less than 1 in dataset 5, and performed poorly in other datasets;
- RLCM achieved the highest TPR in dataset 2, and performed poorly in other datasets;
- TLLCM achieved the highest TPR in dataset 2, TPR greater than 0.9 and less than 1 in dataset 8, while performing poorly in other datasets;
- VAR_DIFF achieved the highest TPR in datasets 2 and 4, TPR greater than 0.8 and less than 1 in datasets 1, 7, 8, and 9, and performed poorly in other datasets;
- ADMD achieved the highest TPR in dataset 7, while performing poorly in other datasets;
- WSLCM achieved the highest TPR in datasets 2, 4, 6, 7, and 8, and the TPR was greater than 0.8 and less than 1 in datasets 1, 3, 5, and 9;
- The proposed algorithm achieved the highest TPR in all nine datasets.

Obviously, the proposed algorithms achieved satisfactory results, but the existing algorithms were affected by varying degrees of background clutter, resulting in algorithm instability. Compared with existing algorithms, the proposed algorithm was more stable, could effectively handle different scenarios, and had the best detection performance.

Table 4 reports the full specification of the implementation environment. The mean runtime was used to demonstrate the computational complexity of different detection algorithms. As can be seen in Table 5, the VAR_DIFF algorithm was faster than other existing algorithms, and the proposed algorithm was second only to the VAR_DIFF algorithm. Although our method was not the most time efficient, it was still relatively fast.

It can be seen from all the above experimental results that none of the existing algorithms could preserve the shape characteristics of the target well. Among them, LCM diffused the target, RLCM diffused the smaller scale target; and LCM, MPCM, RLCM, and TLLCM had poor background suppression. Although VAR_DIFF, ADMD, and WSLCM had strong background suppression capabilities, the detection rate of ADMD was average, and VAR_DIFF and WSLCM had missed detection in scenes with low SCR targets. Although only WSLCM had a relatively low false-alarm rate among existing algorithms, the detection of the WSLCM algorithm is particularly time-consuming; however, the proposed algorithm can preserve the target shape well, has strong background suppression ability, high detection rate, low false-alarm rate, and faster detection speed. In general, the proposed algorithm can effectively preserve the shape features of targets of different scales and types, and can adapt to detection in different scenarios, which further guarantees the speed of the algorithm based on effective detection. Therefore, the proposed algorithm performs better overall.

**Figure 5:**
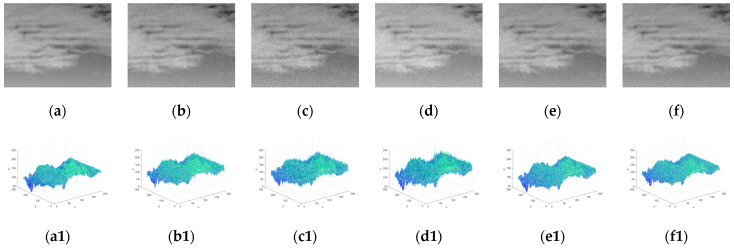
(**a**) Representative images of raw IR dataset 3. (**b**) Gaussian white noise. (**c**) Poisson noise. (**d**) Rayleigh noise. (**e**) Multiplicative noise. (**f**) Uniform noise. (**a1**–**f1**) The 3D gray distribution maps of (**a**–**f**).

**Figure 6:**
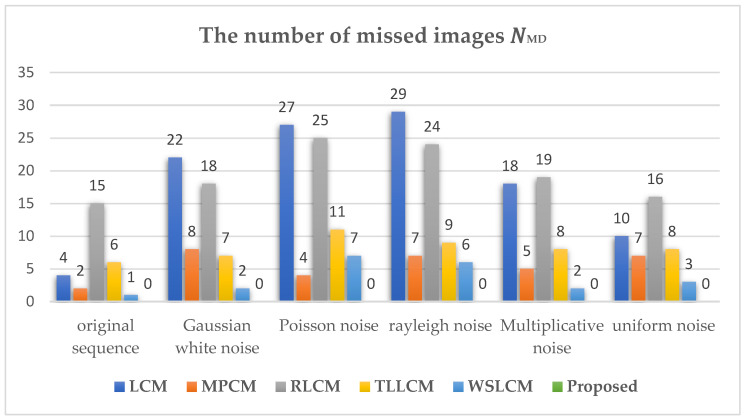
The number of missed images in different algorithms under the influence of noise.

**Figure 7:**
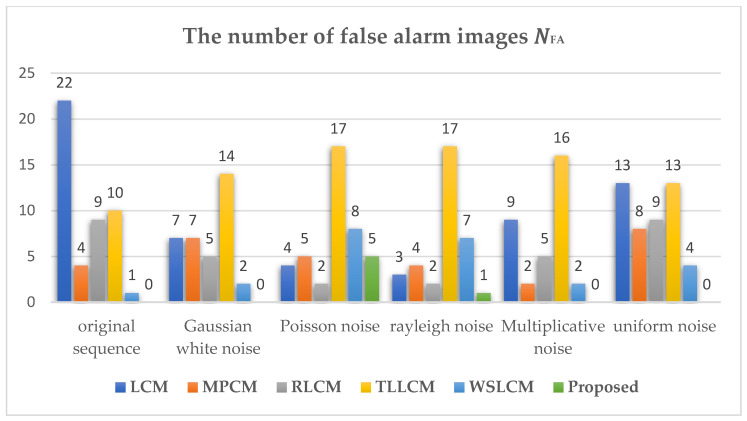
The number of false-alarm images in different algorithms under the influence of noise.

Furthermore, to evaluate the robustness of the proposed algorithm against noise, different types of noise were added to dataset 3 which already contained different degrees of noise for performance comparison. Figure 5 shows representative images of the original IR dataset 3 and images with different types of noise added. Five types of noise were added in the experiment, including Gaussian white noise with a variance of 0.001, Poisson noise, Rayleigh noise with a variance of 15, multiplicative noise with a variance of 3, and uniform noise with a minimum of −14 and a maximum of 14. Table 6 shows the adaptive threshold calculation formulas corresponding to different algorithms and the range of experimental constants. VAR_DIFF and ADMD do not give specific threshold formulas, so the other 5 algorithms were selected for comparison, and the middle value of the applicable range of the constant was used as the constant value in the experiment, the specific information is shown in Table 6. Figure 6 and Figure 7, respectively, show the number of missed images and the number of false-alarm images after different detection algorithms pass the corresponding threshold operation under different noise-type datasets. The experimental results show that the proposed algorithm did not miss detection under the influence of different types of noise. Although the proposed algorithm had false alarms under the influence of Poisson noise and Rayleigh noise, other algorithms had missed detection and false positives under the influence of different noises. Overall, compared with other methods, the proposed algorithm could successfully suppress most of the noise and had strong robustness against noise.

## 4. Conclusions

This paper proposes an IR small-target detection algorithm based on WLDVM. The proposed algorithm performs preprocessing operations through the idea of matched filtering, which can reduce noise and enhance small targets to a certain extent. The distribution characteristics of the target area are fully utilized to divide the window area, which can adapt to the detection of small targets of different scales. LDVM can more effectively highlight the target area and eliminate the bright background, thereby effectively improving the detection rate and reducing the false-alarm rate. The weighting function can improve the adaptability to complex backgrounds and can preserve the shape features of targets of different scales. The fused results can further reduce the missed-detection rate and false-positive rate in complex scenes, thus achieving strong robust detection. Experiments show that the algorithm has good anti-noise ability and is robust to objects of different scales and categories under complex backgrounds. Compared with other methods, the proposed method has obvious advantages in quantitative results such as BSF, SCRG, and the mean runtime, and can better preserve the shape features of targets visually. In future work, we will further study the application of this method in the recognition of IR target categories such as tanks, warships, and aircraft.

## Figures and Tables

**Table 1:** Details of the nine datasets.

	Number of Images	Image Size	Target Size	Target Number	Dataset Type	Target Detail	Background Detail
Dataset 1 [38]	170	250×250	6×6	1	Real sequence	Point target, incomplete occlusion	Complex clouds, complex background
Dataset 2 [39]	429	250×250	6×6	1	Simulated sequence	Point target, low contrast	Heavy noise, dim background
Dataset 3 [5]	30	256×200	4×6 to 5×8	1	Real sequence	Fast-moving, low contrast	Heavy noise, dense clouds
Dataset 4 [40]	39	256×200	4×14 to 7×15	1	Real sequence	Aircraft target, fast-moving	Changing background, thin clouds
Dataset 5 [41]	50	302×202	4×8	1	Simulated sequence	Point target, incomplete occlusion	Remaining almost the same Thin clouds
Dataset 6 [41]	50	238×158	4×8	1	Simulated sequence	Point target, fast-moving	Heavy noise, complex background
Dataset 7 [41]	50	256×239	4×8	1	Simulated sequence	Point target, low contrast	Multiple buildings, heavy noise
Dataset 8 [42]	100	256×256	5×5	1	Simulated sequence	Point target, continuously moving	Heavy noise, land background
Dataset 9 [43]	152	Variety	3×3 to 11×11	1	Non-sequential	Variety	Variety

**Table 2:** The number of false-alarm images $N_{FA}$ and the number of missed images $N_{MD}$ of different datasets under different λ values.

$N_{FA}$	**Dataset**	λ=0	λ=0.1	λ=0.2	λ=0.3	λ=0.4	λ=0.5	λ=0.6	λ=0.7	λ=0.8	λ=0.9	λ=1
	1	**68**	**7**	**4**	**3**	**1**	0	0	0	0	0	0
	2	**130**	0	0	0	0	0	0	0	0	0	0
	3	**30**	**3**	**1**	**1**	0	0	0	0	0	0	0
	4	**39**	0	0	0	0	0	0	0	0	0	0
	5	**50**	**2**	0	0	0	0	0	0	0	0	0
	6	**50**	**11**	**3**	0	0	0	0	0	0	0	0
	7	**50**	**30**	0	0	0	0	0	0	0	0	0
	8	**100**	**100**	**57**	**14**	**4**	0	0	0	0	0	0
	9	**125**	**20**	**6**	**2**	**1**	0	0	0	0	0	0
$N_{MD}$	1	0	0	0	0	0	0	0	0	**37**	**118**	**170**
	2	0	0	0	0	0	0	0	0	**40**	**294**	**429**
	3	0	0	0	0	0	0	0	**3**	**8**	**24**	**30**
	4	0	0	0	0	0	0	0	0	**5**	**27**	**39**
	5	0	0	0	0	0	0	0	**1**	**9**	**24**	**50**
	6	0	0	0	0	0	0	0	0	0	**27**	**50**
	7	0	0	0	0	0	0	0	0	**3**	**29**	**50**
	8	0	0	0	0	0	0	0	**6**	**54**	**92**	**100**
	9	0	0	0	0	0	0	0	**11**	**32**	**83**	**152**

**Table 3:** SCRG and BSF of different detection algorithms.

SCRG	**Dataset**	**LCM**	**MPCM**	**RLCM**	**TLLCM**	**VAR_DIFF**	**ADMD**	**WSLCM**	**Proposed**
	1	0.6001	2.0229	0.7273	2.2439	28.3396	25.9264	26.8277	**44.0452**
	2	0.3256	2.2193	0.6574	1.6246	14.1093	11.7270	25.3549	**27.8967**
	3	0.6322	1.0671	0.7903	1.0808	0.9182	16.6085	30.5386	**105.4981**
	4	0.2055	0.2958	0.2443	0.8030	6.9673	0.3418	3.8525	**18.2806**
	5	0.8549	0.6388	0.7918	0.8555	4.5399	23.1745	21.2844	**51.1140**
	6	0.7983	1.1333	0.9134	1.2898	5.3268	21.8810	**52.8131**	38.8965
	7	0.3171	1.6963	0.4042	0.7671	1.4541	3.2991	**15.9780**	9.5035
	8	0.4021	3.4164	0.6887	1.5680	2.5136	10.5827	30.0565	**39.5189**
	9	0.3886	1.8286	0.7166	1.9683	**24.2053**	12.2716	21.7523	23.6554
	Mean	0.5027	1.5909	0.6593	1.3557	9.8193	13.9792	25.3842	**39.8232**
BSF	1	0.3185	1.6783	0.8350	0.6472	115.0330	12.6716	372.7078	**1144.7466**
	2	0.9805	3.6617	2.0815	1.7074	212.7321	17.2603	758.0712	**3894.0449**
	3	2.1748	6.4061	2.3868	1.8011	**38.5715**	23.4905	37.8834	35.9936
	4	2.2214	11.2676	5.7276	3.3599	100.8143	28.1395	**3704** **.5536**	572.1345
	5	0.5851	2.7340	1.3340	0.8581	30.9868	21.4587	125.5700	**151.6036**
	6	1.0318	4.0402	1.3912	1.1269	22.3591	105.0573	**165.3070**	89.8998
	7	0.4319	1.8672	0.6975	0.5215	8.7144	14.0430	**144.6582**	18.4970
	8	1.4764	6.7578	2.5375	2.3804	29.9505	22.9731	**241.0814**	38.1197
	9	1.4103	8.2744	3.5525	2.5970	974.0334	117.4275	**2401.4376**	2206.5812
	Mean	1.1812	5.1875	2.2826	1.6666	170.3550	40.2802	883.4745	**905.7357**

**Table 4:** The implementation environment.

Operating System	Windows (Windows 10 21H1, x64)
MATLAB version	MATLAB R2020a
CPU	Intel Core i7-10875H @ 2.30 GHz
Memory	16.0 GB

**Table 5:** Comparison of the mean runtime of different algorithms.

Method	LCM	MPCM	RLCM	TLLCM	VAR_DIFF	ADMD	WSLCM	Proposed
Time (s)	0.0274	0.0312	1.1384	0.3216	**0.0068**	0.0167	1.4780	**0.0153**

**Table 6:** Threshold calculation formulas of different algorithms.

	Formula for Threshold Calculation	Range of Experimental Constant	Constant Value Used in the Experiment
**LCM** [5]	$Th=\mu_{SM}+k\sigma_{SM}$ $\mu_{SM}:\;$Mean of SM $\sigma_{SM}:\;$SM standard deviation k: Experimental constant	k∈[3,5]	k=4
**MPCM** [28]		k∈[3,14]	k=8.5
**RLCM** [26]		k∈[2,9]	k=5.5
**TLLCM** [29]	$Th=\lambda max_{SM}+\left(1-\lambda \right)mean_{SM}$ $max_{SM}$: Maximum of SM $mean_{SM}$: Mean of SM λ: Experimental constant between 0 and 1	λ∈[0.7,0.9]	λ=0.8
**WSLCM** [35]		λ∈[0.6,0.9]	λ=0.75
**Proposed**		λ∈[0.5,0.6]	λ=0.55

## Data Availability

Not applicable.
